# Cases in Practice

**Published:** 1887-11

**Authors:** I. P. Wilson

**Affiliations:** Burlington, Iowa.


ARTICLE XI.

### CASES IN PRACTICE.

BY I. P. WILSON, BURLINGTON, IOWA.

CASE I.—Mrs. C., wife of a clergyman, came with her physician to consult me as to the possibility of diseased teeth causing the general debility and nasal catarrh from which she was suffering. The catarrhal symptome had existed for about twelve years; recently her general health was rapidly being impaired, and the catarrh greatly increased. Her breath was intolerably offensive, appetite gone, felt languid and despondent. I found the second left upper molar slightly discolored, but apparently in good condition. No sensitiveness on percussion, but the color of the tooth denoted a dead pulp. From a sense of heaviness in the left cheek, the catarrhal discharge from the left nostril, especially when lying on the right side. I diagnosed the case as one of disease of the antrum, the primary cause being found in the discolored tooth. I accordingly drilled into the pulp chamber and found it filled with putrescent matter confirming my diagnosis. The lady living at a distance I decided to extract the tooth, and was disappointed in not finding pus follow its removal. A probe was readily passed into the antrum and I found the floor thickly covered with a cheesy looking substance, resembling dried pus. Taking a rubber bulb syringe filled with warm water, I thoroughly washed the cavity, the fluid readily passing out of the nose carrying with it large quantities of the purulent matter. I then used, thoroughly, a wash composed of a 5 per cent. solution of carbolic acid, and ordered the treatment continued. After her return home my directions were faithfully carried out by her physician, who reports her rapid return to health. Two months after this operation the husband called to see me, and reported his wife as being in the best of health.

CASE II.—Mrs. R., widow, æt. 30, called to have a tooth filled. Found her suffering from a nasal catarrh of sixteen years standing. Found second bicuspid and third molar on left side above slightly sensitive on percussion with indications of dead pulps; space between them occupied with artificial teeth on rubber plate. She had been under treatment (medically) in Chicage, New York and elsewhere without benefit. Health of late years gradually failing. Breath exceedingly offensive. It had never been suggested to her that diseased teeth might possibly be the cause of her ill-health, and she was loath to follow my advice. Finally she consented, and extracting the third molar I opened into the antrum. An injection of hot water was followed by a gush of corruption from the nose. The treatment of this case with injections of carbolic acid, sulphate of zinc, sulphate of hydrastis, peroxide of hydrogen, was continued for eight months, being used at various times. The discharge gradually subsided, and her health materially improved, when she left for a distant city. I should also state that the second bicuspid was afterwards extracted and found to be in the same condition as the third molar; root much enlarged from excementosis, and the pulp dead. The crowns of both teeth were sound. The tediousness of the treatment was doubtless due to necrosis of the spongy bones around the natural opening from the antrum into the middle meatus of the nose. These bones gradually softened and being disorganized little by little were washed away. The septum of the nose was also perforated, throwing the two passages into one.

It will be remembered that the roots of the superior molars not unfrequently penetrate the maxillary sinus, leaving nothing but the mucous membrane which lines that cavity to protect the apices of the voots. When an abscess forms under such circumstances the discharge will always be into the antrum, and the alveolo-dental membrane will frequently not suffer any serious disturbance, making diagnosis in such cases difficult.

CASE III.—Mrs. B., aet. 23, of robust health, called to have roots of first lower molar extracted. She expressed some doubts about the operation because of a "running sore" on her neck. On examination I found a fistulous opening over the clavicle near the place of origin of the platysma myoides muscle. I had no difficulty in tracing the sinus to the lower border of the jaw directly under the diseased roots. I extracted them and, without any further tteatment, the discharge soon ceased, and abscess healed. The case was of eight years standing and had been pronounced by a counsel of physicians to be of a strumous character. Upon inquiry I found that this tooth had ached just before the swelling appeared on her neck, at which time it ceased aching, and subsequently the crown had broken off leaving the roots in the condition I found them. The facts in the case must have been simply these: The roots of the tooth were unusually long, and when an outlet was sought for the discharge of pus, the weakest point proved to be at the base, instead of the side of the jaw. When nature had secured an outlet through the bone, the ous gravitated down the fibres of the muscle, until it found an outlet at its origin.

CASE IV.—Mr. H., aet. 25, in good health called at my office with a friend. He informed me that he had given up a contemplated trip to his ranch in Texas on account of a malignant growth upon his face. The trouble had been pronounced cancerous, and he had been advised to submit to a surgical operation, as the only hope for his life. I found an irritable looking tumor about the size of a hazelnut immediately below the molar bone. There was no opening in it at the time, but he informed me that slight discharges took place at times. After using the lancet a probe was readily passed through the soft tissues and the alveolar process to the apex of the roots of the first molar. After extracting them and using the carbolic acid wash, I told him he need not hesitate taking his trip, as the cause being removed a rapid cure would result. He has since reported his entire recovery.

The above cases as well as others which have come under my observation, impress me the more that in many forms of obscure trouble about the head and face, the teeth as a probable cause should be carefully inspected—*Archives of Dentistry.*

---

